# Effect of COVID-19-Associated Lockdown on Patients With Diabetic Retinopathy

**DOI:** 10.7759/cureus.14831

**Published:** 2021-05-04

**Authors:** Irini Chatziralli, Eleni Dimitriou, Dimitrios Kazantzis, Genovefa Machairoudia, Georgios Theodossiadis, Panagiotis Theodossiadis

**Affiliations:** 1 2nd Department of Ophthalmology, National and Kapodistrian University of Athens School of Medicine, Athens, GRC; 2 2nd Department of Ophthalmology, National and Kapodistrian University of Athens, Athens, GRC

**Keywords:** covid 19, diabetic retinopathy, lockdown, injections, pandemic

## Abstract

Purpose

To evaluate the effect of the coronavirus disease 2019 (COVID-19)-related lockdown in the management of patients with diabetic retinopathy (DR), including diabetic macular edema (DME), in a tertiary reference center in Greece*.*

Methods

In this retrospective study, we first compared the number of patients who were diagnosed with DR or DME in our clinic during the period of the lockdown and during the same period of the previous year. In addition, we included consecutive patients with DR or DME, who were followed up and treated regularly in our clinic and their appointments deferred due to lockdown, so as to compare the visual acuity, fundoscopy, and optical coherence tomography (OCT) findings prior to and post lockdown.

Results

During the lockdown period, there was a statistically significant decrease in patients with DR and DME as compared to the same period in the previous year. Regarding patients with previously diagnosed DME, there was a statistically significant worsening in their visual acuity and central retinal thickness after lockdown as compared to the last visit before lockdown (p<0.001 for both comparisons). Concerning patients diagnosed with DR and without DME before lockdown, 30% of patients with severe non-proliferative diabetic retinopathy (NDPR) and 8.3% of patients with quiescent proliferative DR (PDR) progressed to active PDR while four out of 107 patients (3.7%) developed DME during the lockdown. Multivariate regression analysis revealed that only the time interval between the last visit before lockdown and the first visit after the lockdown was associated with the best-corrected visual acuity (BCVA) change (p=0.017).

Conclusions

The COVID-19-related lockdown was related to the postponement in patient care, which resulted in significantly worse visual acuity outcomes in patients with DR.

## Introduction

In December 2019, about one year ago, 27 cases of pneumonia of unknown etiology were identified in Wuhan City, China [1]. Noticeably, Dr. Li Wenliang, an ophthalmologist, first recognized the symptoms of severe acute respiratory syndrome coronavirus 2 (SARS-CoV-2, now known as coronavirus disease 2019 (COVID-19)) in seven of his patients while he developed the disease himself and passed away in February 2020 [2]. The outbreak of COVID-19 was sudden and unprecedented since its spread was very quick and extensive; therefore, the World Health Organization (WHO) has declared COVID-19 as a pandemic [3].

The pandemic changed the whole world. Since social distancing is a key measure to slow the transmission of the virus, in many countries, governments decided to implement lockdowns [4-5]. Although necessary to control the pandemic, these measures have resulted in major interruptions in the economy, social life, and healthcare provision [4]. Specifically, hospitals have altered general wards into intensive care units (ICUs), reduced outpatient clinics, canceled elective surgeries, and re-deployed healthcare providers while treatment has been restricted to urgent or emergency conditions [4-5].

In Greece, a partial lockdown was initiated on March 16, 2020, and became a complete lockdown on March 23, 2020, lasting until May 10, 2020. During the lockdown, the national healthcare system focused on the prevention and management of COVID-19-related disease and emergency services only. In the majority of ophthalmology clinics in Greece, regular clinic visits, elective surgeries, scheduled intravitreal injections, and non-urgent eye conditions were deferred, as it also occurred in most countries worldwide [4-8]. In addition, many patients have themselves postponed their visits to ophthalmology clinics in order to avoid being exposed.

Diabetic retinopathy (DR) is a microvascular complication of diabetes mellitus and one of the leading causes of blindness in the working-age population, especially due to the development of diabetic macular edema (DME) or proliferative DR (PDR), both of which require prompt management and regular follow-up [9-10]. The standard of care for DME is anti-vascular endothelial growth factor (anti-VEGF) injections, which have been shown to be safe and effective in large pivotal clinical trials [11-12]. Accordingly, panretinal photocoagulation (PRP), anti-VEGF injections, or a combination of them are used today for the treatment of PDR [13-16] before ending up in advanced-stage disease, including vitreous hemorrhage (VH) and tractional retinal detachment (TRD) [10,17]. It is worthy to note that failure to visit clinicians and to undergo the appropriate treatment may result in worse outcomes and potential irreversible visual loss in patients with DR [18-19].

In light of the above, the purpose of this study was to evaluate the effect of COVID-19-related lockdown in the management of patients with DR in a tertiary reference center for “diabetic eye disease” in Greece. We hope that this analysis will provide valuable insight into the management of patients with DR in real-life emergency settings, such as another wave of COVID-19 outbreak or other future pandemics.

## Materials and methods

Participants in this retrospective observational study were patients who attended the “diabetic eye clinic” or the emergency ophthalmology department with a diagnosis of DR, DME, or VH/TRD due to PDR during the COVID-19-related lockdown and during the same period in the previous year at a tertiary reference center in Greece (2nd Department of Ophthalmology, National and Kapodistrian University of Athens, Athens, Greece). In addition, the data of patients, who were regularly followed up and treated for DME or DR in the “diabetic eye disease” clinic of our department and were supposed to attend the clinic, but deferred due to the COVID-19-related lockdown and were examined after the lockdown, were collected. The study was in adherence with the Declaration of Helsinki and no approval by the Institutional Review Board of our hospital was needed since it was a retrospective study. Informed consent was obtained from participants in this study.

For all participants, their medical records were reviewed and analyzed. Demographic data, medical history, dates when they attended clinics and received treatment, as well as clinical data regarding best-corrected visual acuity (BCVA; Snellen charts), dilated fundoscopy findings, and optical coherence tomography (OCT) assessments were recorded. OCT had been performed in all patients using the Heidelberg Spectralis HRA+OCT device (Heidelberg Engineering, Heidelberg, Germany).

Statistical analysis was done using the Statistical Package for Social Sciences (version 24.0, IBM Corp, Armonk, NY). Comparisons between the two years were performed using the student’s t-test or Mann-Whitney-Wilcoxon test, as appropriate. Accordingly, comparisons for the same patients between the periods before and after lockdown were performed using the paired sample t-test or the Wilcoxon signed-rank test. Qualitative variables were assessed using the chi-square test. The association between the change in BCVA between the last visit before lockdown and the first visit after lockdown (dependent variable) and other demographic and clinical characteristics of patients (independent variables) was evaluated using multivariate regression analysis. Statistical significance was set as a p-value of <0.05.

## Results

During the complete lockdown period, we did not run the retina and the “diabetic eye disease” clinics, and all regularly scheduled visits were deferred. No intravitreal injections were performed in our hospital. We only accepted emergencies and performed urgent surgeries, always using suitable personal protective equipment and measurements. Specifically, six patients visited the emergency department due to PDR with only neovascularization, eight patients due to VH or TRD, and seven patients due to DME, while five patients were diagnosed with non-proliferative DR (NPDR). Regarding treatment, four patients received PRP and one patient underwent PPV for TRD. Table 1 shows the data during the same period in 2019 and 2020. As compared to the previous year, there is a significant decrease in patients diagnosed with both NPDR and PDR with only neovascularization, as well as in patients with DME, while there was no statistically significant difference in patients with VH or TRD who attended the emergency department. Accordingly, there was a statistically significant decrease in the number of intravitreal injections, PRP sessions, and PPV for the treatment of PDR complications performed in our department during the lockdown period as compared to the same period of the previous year.

**Table 1:** Data regarding the number of patients with diabetic retinopathy diagnosed and treated during the same period (March 23-May 10) in 2019 and 2020 (lockdown) VEGF: vascular endothelial growth factor

	2019	2020
Non-proliferative diabetic retinopathy	183	5
Proliferative diabetic retinopathy (only neovascularization)	21	6
Vitreous hemorrhage or tractional retinal detachment	9	8
Diabetic macular edema	147	7
Intravitreal anti-VEGF injections	132	0
Intravitreal steroids injections	7	0
Panretinal photocoagulation	18	4
Pars plana vitrectomy	6	1

In this study, we also included 62 consecutive patients with previously diagnosed DME and 107 patients with DR who were supposed to be examined and treated during the lockdown since they were regularly followed up in our department, but their appointments were deferred due to the COVID-19-related lockdown.

**Figure 1:**
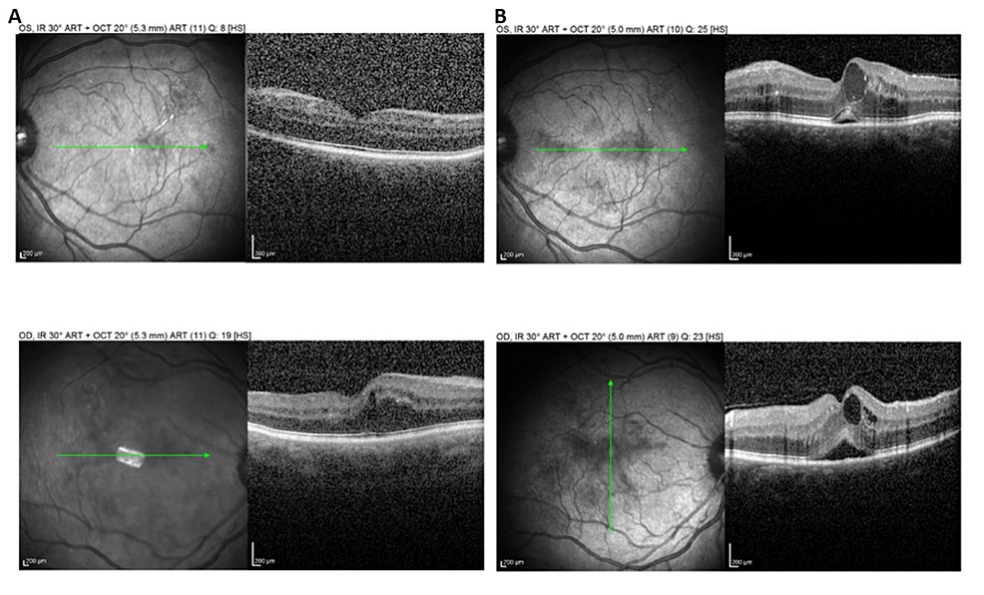
(A) Structural optical coherence tomography (OCT) of a male patient with diabetic macular edema in the right eye (bottom panel) at the last visit before lockdown. Note that there was no macular edema in the left eye (top panel). Visual acuity was 0.7 and 0.9 decimal in the right and left eye, respectively; (B) Structural OCT from the same patient at the first visit after the lockdown, where there was worsening of macular edema in both eyes with serous retinal detachment and cystoid spaces in both eyes. The visual acuity was 0.5 decimal in both eyes.

Regarding patients with previously diagnosed DME, the mean BCVA at the last visit before the lockdown was 0.42±0.14 (decimal scale) and differed significantly compared to the mean BCVA at the first visit after the lockdown (0.27±0.17, p<0.001). Of note, 11 out of 62 patients with DME (17.7%) presented a very low BCVA of ≤0.1 decimal prior to the lockdown while after the lockdown, 21 out of 62 patients (33.9%) presented BCVA ≤0.1 decimal, a difference that was statistically significant (p=0.040). Accordingly, the mean central retinal thickness (CRT) at the last visit before the lockdown was 379.3±51.9 μm and differed significantly with the CRT at the first visit after lockdown (481.4±69.2 μm, p<0.001). Since no intravitreal injections were administered for patients with DME/DR during the lockdown period, there was a delay in attending their original intravitreal injection appointment of 8.2±2.3 weeks. Figure 1 shows a case of our study sample, presenting worsening of DME in both eyes after lockdown.

As far as patients with previously diagnosed DR without DME, there was no statistically significant difference in BCVA at the first visit after lockdown compared to the last visit before lockdown (0.64±0.13 vs. 0.70±0.09 decimal scale, respectively, p=0.059). Table 2 shows the progression in disease stage at the first visit after the lockdown compared to the last visit before the lockdown. Specifically, one patient progressed from mild to moderate NPDR and one from moderate to severe NPDR. However, three out of 10 patients with severe NPDR (30%) progressed to active PDR, one of whom developed vitreous hemorrhage during the lockdown period while one patient with previous quiescent PDR (8.3%) exhibited active PDR again. Additionally, four out of 107 patients (3.7%) developed DME during the lockdown period. 

**Table 2:** Progression in disease stage after lockdown compared to prior lockdown in patients with diabetic retinopathy and without macular edema. PDR: proliferative diabetic retinopathy; NPDR: non-proliferative diabetic retinopathy

	Last visit before lockdown	First visit after lockdown
Mild NPDR	51	50
Moderate NPDR	26	26
Severe NPDR	10	8
PDR quiescent	12	11
PDR with active neovascularization	7	10
PDR with vitreous hemorrhage or tractional retinal detachment	1	2

The results of the multivariate regression analysis are shown in Table 3. Only the time interval (in weeks) between the last visit before the lockdown and the first visit after the lockdown was found to be associated with BCVA change, with a longer interval to be associated with worse visual acuity (β coefficient=0.451, p=0.017) while there was a trend for the DR stage, which did not reach statistical significance (p=0.052).

**Table 3:** Results of multivariate regression analysis for the association between the change in visual acuity at the first visit after the lockdown compared to the last visit before the lockdown and the clinical characteristics of the study sample. BCVA: best-corrected visual acuity; CRT: central retinal thickness

	β coefficient	p-value
Age (years)	0.314	0.115
Gender (male vs female)	0.108	0.259
Number of previous intravitreal injections	0.094	0.509
BCVA at the last visit before lockdown (decimal)	0.017	0.642
CRT at the last visit before lockdown (μm)	0.083	0.091
Diabetic retinopathy stage at the last visit before lockdown	0.299	0.052
Time interval between the last visit before lockdown and the first visit after lockdown (weeks)	0.451	0.017

## Discussion

The principal message of this study was that a COVID-19-related lockdown has a negative impact on patients with DR, including DME. First of all, there was a significant decrease in the number of patients with DR who visited our clinic during the lockdown period as compared to the same period during the previous year, as was seen in previous studies as well [4,6-7,20]. Moreover, patients with previously diagnosed DR and DME could not attend the clinic due to lockdown since all regular follow-up visits and intravitreal injections appointments deferred. This resulted in a significant worsening in BCVA and CRT in patients with DME, as well as in progression to active PDR in 30% of patients with severe NPDR and in 8.3% of patients with previously quiescent PDR. Our findings seem to be independent of glycemic control since diabetic patients were found to exhibit a small but significant improvement in glycemia, body weight, and total cholesterol while the other metabolic parameters remained stable [21].

Several organizations have published general guidance for ophthalmologists on managing patients during the pandemic, including the American Academy of Ophthalmology, the French Society of Ophthalmology, the German Ophthalmological Society, and the Royal College of Ophthalmologists [22-25] while the Vision Academy Steering Committee has provided specific guidance for intravitreal anti-VEGF injections during the COVID-19 pandemic [26]. All these guidelines conclude that strategies for managing patients with a retinal disease during this uncertain time should focus on minimizing the risk of exposure to COVID-19 for both patients and healthcare staff, prioritizing treatment for those at greatest risk of irreversible vision loss, and simplifying anti-VEGF treatment regimens [26-27]. Overall, there is a general consensus that protective measures should be applied in ophthalmic practice due to the increased risk for transmission of COVID-19 with the close proximity that is often required between physicians and patients. Additionally, risk assessment, postponement of non-urgent cases, and teleophthalmology virtual services have been proposed [26].

Regarding patients with DR or DME, the Royal College of Ophthalmologists guidelines recommend deferring anti-VEGF injections and reviewing them in the clinic after four months, with the exception of patients with severe NPDR and active PDR, who may require anti-VEGF agents and PRP, while a virtual review with OCT and wide-field color photography seems to be the preferred option to review these patients [25]. However, as the Vision Academy Steering Committee underlined, these guidelines are specifically relevant to the UK healthcare system and application outside the UK may be confounded by local regulations, practice capacities, and other country-specific factors [26].

Anti-VEGF therapy for patients with DME needs ongoing, perpetual treatment in most eyes. Therefore, suspending intravitreal injection appointments or examination of patients with DME may result in a significant visual loss in some patients [28], as was observed in our study as well. The same applies to patients with severe NPDR or active PDR, who should receive their treatment at the earliest convenience, to avoid the devastating complications of PDR. Since our results showed that delays in treatment were associated with a change in BCVA, prolonged treatment postponement and indefinite deferral of the appointments without rescheduling within a reasonable time should be avoided, depending on measures in each individual country [26,28].

Previous studies have shown similar results in patients with neovascular age-related macular degeneration during the COVID-19-related lockdown. Specifically, Borelli et al. found that the COVID-19 pandemic resulted in a significant delay in neovascular age-related macular degeneration patient care, which was associated with worse short-term outcomes in these patients, suggesting more flexible treatment regimens in patients, who need frequent treatment [6].

A potential limitation of our study pertains to its retrospective nature, as selection bias could be anticipated. Moreover, we did not include data about modifiable factors, such as hypertension, kidney disease, anemia, or glycemic status, which could affect DR. In addition, the study sample seems to be relatively small and derived from a single center. However, this is a real-life study, investigating the impact of a COVID-19-related lockdown in patients with DR at a tertiary reference center for “diabetic eye disease.”

## Conclusions

In conclusion, to our knowledge, this is the first study in Greece evaluating the impact of a COVID-19-related lockdown in patients with DR, showing that unintentional interruption of follow-up and treatment can result in significant deterioration in visual acuity. Of note, since visual acuity outcomes were found to be associated with the time interval between the visits before and after lockdown, reflecting the length of treatment interruption, prolonged postponement of examination or treatment should be avoided. Our duty is to balance the desire to treat our patients while protecting them from being harmed by inadvertent viral transmission. Since the COVID-19 pandemic is still in progress and other global hazards may occur with restrictions in healthcare groundwork, our study provides useful information about the management of patients with DR in real-life clinical practice. Long-term studies are needed to expand our results and suggest guidelines for individualized treatment.

## References

[1] Lu H, Stratton CW, Tang YW (2020). Outbreak of pneumonia of unknown etiology in Wuhan, China: the mystery and the miracle. J Med Virol.

[2] Parrish RK 2nd, Stewart MW, Duncan Powers SL (2020). Ophthalmologists are more than eye doctors—in memoriam Li Wenliang. Am J Ophthalmol.

[3] Cucinotta D, Vanelli M (2020). WHO declares COVID-19 a pandemic. Acta Biomed.

[4] Saleh OA, Jammal H, Alqudah N, Alqudah A, Abu-Yaghi N (2020). Clinical experience in the administration of intravitreal injection therapy at a tertiary university hospital in Jordan during the COVID-19 lockdown. Clin Ophthalmol.

[5] Chatziralli I, Ventura CV, Touhami S (2021). Transforming ophthalmic education into virtual learning during COVID-19 pandemic: a global perspective. Eye (Lond).

[6] Borrelli E, Grosso D, Vella G (2020). Short-term outcomes of patients with neovascular exudative AMD: the effect of COVID-19 pandemic. Graefes Arch Clin Exp Ophthalmol.

[7] dell'Omo R, Filippelli M, Semeraro F (2020). Effects of the first month of lockdown for COVID-19 in Italy: a preliminary analysis on the eyecare system from six centers. Eur J Ophthalmol.

[8] Yang KB, Feng H, Zhang H (2020). Effects of the COVID-19 pandemic on anti-vascular endothelial growth factor treatment in China. Front Med (Lausanne).

[9] Forbes JM, Cooper ME (2013). Mechanisms of diabetic complications. Physiol Rev.

[10] Antonetti DA, Klein R, Gardner TW (2012). Diabetic retinopathy. N Engl J Med.

[11] Mitchell P, Bandello F, Schmidt-Erfurth U (2011). The RESTORE study: ranibizumab monotherapy or combined with laser versus laser monotherapy for diabetic macular edema. Ophthalmology.

[12] Heier JS, Korobelnik JF, Brown DM (2016). Intravitreal aflibercept for diabetic macular edema. 148-week results from the VISTA and VIVID studies. Ophthalmology.

[13] Gross JG, Glassman AR, Liu D (2018). Five-year outcomes of panretinal photocoagulation vs intravitreous ranibizumab for proliferative diabetic retinopathy. A randomized clinical trial. JAMA Ophthalmol.

[14] Sivaprasad S, Prevost AT, Vasconcelos JC (2017). Clinical efficacy of intravitreal aflibercept versus panretinal photocoagulation for best corrected visual acuity in patients with proliferative diabetic retinopathy at 52 weeks (CLARITY): a multicentre, single-blinded, randomised, controlled, phase 2b, non-inferiority trial. Lancet.

[15] Figueira J, Fletcher E, Massin P (2018). Ranibizumab plus panretinal photocoagulation versus panretinal photocoagulation alone for high-risk proliferative diabetic retinopathy (PROTEUS study). Ophthalmology.

[16] Fallico M, Maugeri A, Lotery A (2020). Intravitreal anti-vascular endothelial growth factors, panretinal photocoagulation and combined treatment for proliferative diabetic retinopathy: a systematic review and network meta-analysis. Acta Ophthalmol.

[17] Khan R, Surya J, Rajalakshmi R (2020). Need for vitreous surgeries in proliferative diabetic retinopathy in 10 years follow-up: India Retinal Disease Study (IRDS) group report no. 2. Ophthalmic Res.

[18] Abdelmotaal H, Ibrahim W, Sharaf M, Abdelazeem K (2020). Causes and clinical impact of loss to follow-up in patients with proliferative diabetic retinopathy. J Ophthalmol.

[19] Bresnick G, Cuadros JA, Khan M (2020). Adherence to ophthalmology referral, treatment and follow-up after diabetic retinopathy screening in the primary care setting. BMJ Open Diabetes Res Care.

[20] Wasser LM, Weill Y, Brosh K (2020). The impact of COVID-19 on intravitreal injection compliance. SN Compr Clin Med.

[21] Psoma O, Papachristoforou E, Kountouri A (2020). Effect of COVID-19-associated lockdown on the metabolic control of patients with type 2 diabetes. J Diabetes Complications.

[22] (2021). American Academy of Ophthalmology. Important coronavirus updates for ophthalmologists. https://www.aao.org/headline/alert-important-coronavirus-context.

[23] (2021). Société Française d' Ophtalmologie. Recommandations de la SFO face à l'épidémie au Covid-19 [Website in French]. https://www.sfo.asso.fr/actualites/recommandations-de-la-sfo-face-lepidemie-au-covid-19.

[24] (2021). Deutsche Opthalmologische Gesellschaft. Coronavirus COVID-19 [Website in German]. https://www.dog.org/?cat=288.

[25] (2021). The Royal College of Ophthalmologists. COVID-19 clinical guidance for ophthalmologists. https://www.rcophth.ac.uk/about/rcophth-guidance-on-restoring-ophthalmology-services/rcophth-covid-19-response.

[26] Korobelnik JF, Loewenstein A, Eldem B (2020). Guidance for anti-VEGF intravitreal injections during the COVID-19 pandemic. Graefes Arch Clin Exp Ophthalmol.

[27] Korobelnik JF (2020). Response to letter: COVID-19 and macular edema-a necessary blindness?. Graefes Arch Clin Exp Ophthalmol.

[28] Stone LG, Devenport A, Stratton IM, Talks JS (2020). Macula service evaluation and assessing priorities for anti-VEGF treatment in the light of COVID-19. Graefes Arch Clin Exp Ophthalmol.

